# Smart Technologies Lead to Smart Answers? On the Claim of Smart Sensing Technologies to Tackle Animal Related Societal Concerns in Europe Over Current Pig Husbandry Systems

**DOI:** 10.3389/fvets.2020.588214

**Published:** 2021-01-08

**Authors:** Mona F. Giersberg, Franck L. B. Meijboom

**Affiliations:** Animals in Science and Society, Department Population Health Sciences, Faculty of Veterinary Medicine, Utrecht University, Utrecht, Netherlands

**Keywords:** pig production, precision livestock farming, society, perception, attitude

## Abstract

Current pig production systems in Europe are subject to public criticism. At the same time, Precision Livestock Farming (PLF) technologies, which allow for automated animal monitoring are entering commercial pig farms. With their claim of improving animal health and welfare, these innovations may respond to public concerns. However, they may raise problems of societal acceptance themselves. In this review, we investigate whether the available literature allows for an analysis to which extent PLF can mitigate or reinforce societal concerns related to pig production. We first analyze papers on pig husbandry systems in general, and then those on PLF as an innovation in animal production. In general, there is a tension between citizens and farmers. Citizens hold rather negative attitudes whereas farmers evaluate pig production more positively. Literature on attitudes of other actors, such as veterinarians, is missing. Information on the attitudes toward PLF of stakeholders other than farmers is lacking. Possible challenges of societal acceptance of PLF and chances to overcome these are only discussed in theoretical approaches. We conclude that to analyze the role of PLF in addressing societal concerns over pig production, there is a need for further empirical research including attention to underlying values of all stakeholders. This should focus on the attitudes of the currently missing stakeholders toward pig husbandry in general, and on those of the wider society toward PLF. Only by means of additional data, it will be possible to evaluate whether PLF has the potential to address societal concerns related to pig production.

## Introduction

Societal acceptance of livestock production depends no longer on economic criteria or arguments of food security only (1). Public evaluation focuses also on animal welfare, public health risks, and environmental sustainability. As a result, European pig production, which mainly takes place in intensive indoor systems, and at distance from an increasingly urbanized population, is no exemption to public criticism (1). Although the relationship between public criticism and pork consumption seems to be rather diffuse, critical attitudes communicated in public debate influence policy formation (2). Thus, the sector's future “license to produce” depends on the extent to which it is able to respond to societal concerns (3). Rather than considering these concerns as a hurdle which has to be taken to strive for acceptance, it can be also seen as a basic driver for innovation (4).

An example of such an innovation is Precision Livestock Farming (PLF). This can be integrated into the current system of livestock production but has also the potential to act as a trigger for substantial system change. PLF is defined as the management of livestock production by means of the principles and technologies of process engineering (5). It relies on automatic monitoring of animals and related environmental processes by various smart sensing technologies. Proponents of PLF promise improved animal health and welfare, increased productivity and the mitigation of the environmental impact of livestock production (6, 7). As is the case with most technological innovation, PLF may face questions of data ownership and privacy (8) and issues of peoples' technology readiness (9). However, PLF is more complex than for instance precision crop farming. In contrast to crops, animals are recognized as sentient beings that can interact and build relationships with humans (10). Animals being subjected to technologies which may replace human care and human-animal relationships might therefore be reason for concern itself. Thus, it is not known to which extent—if at all—PLF may be a means to address societal concerns in relation to current pig production.

The aim of this paper is to investigate whether the available literature allows for an analysis to which extent PLF technologies have the potential to mitigate or reinforce societal concerns related to current pig production. For this aim we explore public attitudes toward pig production systems and toward PLF technologies as have been described and analyzed in literature. We assess the type and direction of reported attitudes and identify chances and challenges with regard to PLF as an innovation to deal with societal concerns over pig production.

## Methods

Literature was retrieved from “Web of Science” applying the search terms “pig husbandry,” “pig production,” and “Precision Livestock Farming” in combination with each of the following terms: “society,” “citizen,” “veterinarian,” “farmer,” “attitude,” “perception,” “moral,” and “ethic.” Peer-reviewed original research and review articles in English published in and after the year 2000 were included. This time frame was chosen as PLF emerged at the beginning of the twenty-first century, with the first European PLF conference realized in the year 2003 (11). Regarding empirical research, only studies conducted in the European Union were considered, as Member States share the same legal framework of minimum standards for pig production (12). Including research from other parts of the world would result in an even larger variation of socio-cultural backgrounds and traditions, whose discussion would go beyond the scope of this paper. References were scanned for topical relevance. Papers which met this criterion were analyzed with focus on stakeholders, aspects of pig production or PLF, output variables (e.g., perceptions, attitudes) and explanatory variables (e.g., demographics).

## From Attitudes To Chances and Challenges of Societal Acceptance: The Case of Current Pig Production Systems

Applying the above-mentioned search criteria resulted in 15 papers on pig production suitable for inclusion. Except for two of them (1, 13), they were empirical studies, eight of which used a quantitative, three a qualitative and two a mixed-method approach. First, we specify the stakeholders who appear in the studies on attitudes toward pig husbandry systems, followed by a description of the methods used and the specific aspects of pig husbandry addressed in these studies. The third paragraph of this section reports the independent and dependent variables, and the way they are analyzed in the reviewed literature. The fourth paragraph presents the results of the most extensively researched stakeholder group, the citizens, regarding the independent variables, followed by a paragraph on the explanatory value of the dependent variables. It is followed by a similarly structured paragraph on the results of the studies which deal with further stakeholders.

Most studies deal with the attitudes of citizens and consumers. In two cases, the terms “consumer” and “citizen” are used synonymously (13, 14). Methodological, this seems unfavorable, as the vegetarians and vegans explicitly included in the study (14) will not see themselves as consumers of pork. On the other hand, it can be argued that consumer concerns are no longer restricted to traditional market or consumption related concerns (15, 16), for instance consumer concerns include also animal welfare issues. These concerns matter to people not only in their role as consumer, but because certain production methods are not compatible with their ideas about the good society (15). Additionally, some papers focus on farmers (17, 18), sometimes with a distinction between conventional and organic pig farmers (19–21). Only two studies deal with further actors, i.e., pig husbandry advisors and veterinarians (21), or speak about producers in general terms [i.e., everyone involved in the chain, ranging from farmers to managers of supermarkets (1)].

The quantitative studies use a predetermined set of items (22–24), statements (2, 24, 25), or pictures (14, 26) which are rated by the participants. Most items address animal-, housing-, and management related aspects of pig welfare. In addition, part of the investigations cover other domains linked to pig production, such as product quality (2, 24), trust in food chain actors (22), public health (24, 25), farmers (24, 25), and environmental impact (2, 24). The content and wording of the items are mostly drawn from literature, in some cases also from previous focus group discussions (23, 25), or expert consultations and media analyses (24). In the only qualitative study on citizens' attitudes, participants are asked for concerning but also for appreciated aspects of pig husbandry after having visited a farm (27). In contrast to evaluating predetermined items, people could indicate concerns over and appreciation for aspects of their own choice (27).

The independent variables measured in the reviewed studies differ in nuance. The predominantly assessed entity is “attitude” toward the above described aspects (2, 18, 21, 22, 24, 26). In addition, some authors (22, 25, 26) investigate “perception,” i.e., the identification, organization, and interpretation of sensory input, as a premise to understand the resulting broader mental and emotional entity of “attitude” (28). Some sources also report output variables that go beyond “attitude.” For instance, the level of acceptance of features of pig husbandry is explored directly (14, 21, 25) or indirectly (23). It is argued that regardless peoples' possible negative attitudes toward a given aspect, they may still find this aspect acceptable within the present situation (21). “Moral values” are studied and are found unsuitable as predictors for attitudes (18), as a different relative weighing and interpretation of the same set of moral values may lead to different attitudes (18). The output variable “concern” is interpreted to simply arise from negative attitudes (18) or conceptualized in more detail as the combination of a negative evaluative belief and a high perceived importance (23). To understand the direction of resulting attitudes, various explanatory variables are studied in the reviewed papers, such as demographics, belonging to a certain stakeholder group, knowledge about agriculture (25), level of meat consumption (2, 14), and belief in mental abilities of pigs (22, 26). Based on the variables mentioned above, data were analyzed to either identify clusters of similar attitudes within one group of stakeholders (2, 14, 22, 24, 25) or to compare different groups (17, 18, 21).

The direction of reported attitudes toward pig husbandry is quite negative: in one study, citizens state that additional care is necessary for all items presented to them (24). Similarly, citizens commonly express concerns about the welfare of pigs in current housing systems (22, 23, 25). In contrast, Krystallis et al. (2) show that people have rather moderate, i.e., neither strongly negative nor strongly positive attitudes toward pig welfare, environmental protection, and industrial food production. On closer inspection, citizens seem to think of pig welfare in two broad categories: animal health (defined by environmental indicators) and suitability of housing conditions (22). This is illustrated by Busch et al. (26) who find that peoples' evaluation of animal welfare is more influenced by their evaluation of the husbandry system a pig is presented in (environmental indicator) than by their interpretation of this pigs' body language (animal-based indicator). In line with this focus on environmental indicators, strong concerns are expressed regarding small pen sizes (23), slatted floor (2) and the absence of outdoor access (2, 18). When asked to evaluate aspects of their own choice, half of the participants mention and appreciate automatization, such as computer registration of animals and automatic feeding (27). Citizens further value good care for the animals, which they define as regular farmer-animals contact and looking after ill pigs. At the same time as they appreciate modernization, citizens have concerns similar to those reported in the quantitative studies above (27). Within the group of consumers-citizens some authors identify different clusters, which are comparable between studies. These clusters are for instance described as “ambivalent/unsure” or “animal welfare conscious/supporters” and relate to the level of concern people express regarding the aspects of pig production presented to them (2, 14, 24).

The explanatory impact of demographic characteristics varies between studies. Bergstra et al. (24) and Krystallis et al. (2) observe that a lower level of education increases the probability to hold more negative attitudes toward pig welfare, whereas Weible et al. (25) find no such effect. The same is true for most other explanatory variables, such as meat consumption (2, 14) or peoples' beliefs in pigs' mental abilities (22, 26). Nonetheless, more knowledge about certain husbandry systems or agricultural practices seems to lead consistently to less acceptance (14, 25).

In contrast to the wider society, farmers evaluate the current state of pig welfare more positively (17, 18, 21). Discordance is highest for the aspects natural behavior, pain, stress, and space availability (17). In the one study that considers various stakeholders (21), these can be grouped based on their attitudes and level of acceptance of aspects of pig husbandry. Citizens and organic pig farmers share negative attitudes, whereas conventional farmers and advisors are positive about most of the aspects presented. A third group is formed by pig veterinarians, who hold a mix of positive and negative attitudes. For instance, veterinarians evaluate indoor housing positively (similar to conventional farmers), whereas they find interventions without sedation inacceptable (similar to citizens). The few qualitative studies on farmers' attitudes point in the same direction: in general, farmers are positive about current pig husbandry and agree that pigs have a good life (19, 20). However, the definition of welfare and the motivation to rear pigs to higher standards than legally required varies among subgroups of farmers. The motivation of organic farmers is based on convictions and for them, good pig welfare includes the possibility to perform natural behavior. Conventional farmers, on the other hand, define welfare as good health and performance, and see the participation in quality schemes as a prerequisite to produce (19, 20).

Three main conclusions can be drawn from the literature analyzed above. First, citizens hold rather negative attitudes mainly toward housing related aspects of pig husbandry. This poses challenges for the societal acceptability of these systems. Second, the direction of criticism is not uniform. Instead, the level of concern varies among different aspects of pig production and subgroups of citizens. Finally, citizens' attitudes are at odds with farmers' more positive evaluations of pig production.

## PLF Technologies: Key To Tackle Concerns or Poser of Intrinsic Objections?

To discuss whether (part of) the public concerns regarding pig husbandry can be tackled by implementing PLF technologies, it is important to know stakeholders' attitudes toward PLF. However, the methods used in the current literature on PLF differ from those applied in the studies analyzed in the previous section. The majority of papers on PLF, which met the inclusion criteria, were non-empirical studies or reviews. Only one study examined farmers' attitudes toward PLF quantitatively (29), whilst another included semi-structured interviews (30). We first present the results of these empirical studies, followed by the findings of the reviews and conceptual papers on PLF.

Lima et al. (29) investigated drivers for farmers to adopt PLF-tools for flock management. Although electronic identification tags for sheep represent a rather unsophisticated technology in a less intensified branch, general conclusions might be applicable to the pig sector. Implementation of PLF is influenced by three main factors: farmers who perceive the tool as “useful” and “practical” deliberately adopt it, whereas farmers who feel under (governmental) pressure to apply technology see the tool as a burden and are more reluctant to use it (29). Farmer characteristics associated with a more positive attitude toward the tool are a higher level of IT knowledge, the use of a smartphone, the intention to intensify production and longer times spent with the flock. On the other hand, farmer age, farm size, and social pressure by other farmers have no effect on the adoption of PLF (29). Hartung et al. (30) interviewed pig farmers who have installed tools for behavior recognition and climate control as part of an on-farm research project. The expected advantages according to the farmers are more stability in production and less routine work. Their concerns are mainly related to the functioning and maintenance of the technology (practicality), and whether the expected benefits can be demonstrated (usefulness). Most farmers have problems with understanding, interpreting and identifying with the generated data. They understand the application as “research project” and they feel left alone with technology and regulations. In addition, they doubt that PLF would raise societal acceptance of livestock production (30).

Reviews or perspectives on the potentials of PLF for sustainable pig husbandry are found to rely on presuppositions and speculations without providing empirical evidence or reasonable explanation. It is for instance assumed that PLF would enhance consumer acceptance of pig production as consumers would appreciate systems that imply individual attention and good human-animal interaction (31). However, it is not argued to why PLF should necessarily and exclusively be a per-animal-approach, and thus lead to better care and welfare for the individual. Similarly, practitioners, defined as veterinarians, animal scientists, and geneticists, could assist farmers to establish standards for responsible data storage and use to tackle privacy and data rights issues (31), although there is no evidence in the curriculum of veterinarians or from their own indications that they are able and willing to fulfill such a role. In another paper, the possibility of real-time welfare monitoring in intensive production systems by building “digital representations” of the animals is postulated to bring them closer to the farmer (32). Unfortunately, the assumed causal relationship of continuous data generation and enhanced attention to the animals is not further elaborated. Finally, it is simply stated that “PLF will provide the license to produce” within the inevitable process of intensification (6).

Conceptual approaches delve deeper into possible opportunities and challenges of PLF with regard to relationship issues (33, 34) and its embeddedness in nature (35). Bos et al. (33) investigate the link between PLF and a further objectification of animals in intensive livestock production. If mainly understood as elements of a system, the animals' integrity would be compromised, which would also be true for the farmers' identity in such a system (33). Whilst PLF could increase the instrumental reasoning of the farmer by pursuit of knowledge (data) and control (interventions), it could at the same time redefine the notion of care, if care for the individual animal is sufficiently implemented in such technologies (33). The authors conclude that moral assessment of PLF is not possible without considering the effects on caring relationships in specific settings (33). Werkheiser (34) applies the parenting analogy to examine whether farmers are using PLF in a way that allows them to discharge their duties of personal responsibility for caring for their animals. Even though that may be the case, PLF will not help farmers to be better farmers in a traditional sense, i.e., the issue of reconciling intensive livestock production with what is perceived as “good farming practice” will remain (34). There seem to be further relationship issues associated with PLF, for instance that PLF replaces positive human-animal interactions, while stressful tasks still have to be performed by humans in then less habituated animals (36). A first approach of a biomimetic conceptualization of PLF as ecological innovation embedded in and in accordance with the natural environment is proposed by Blok and Gremmen (35), to overcome ethical issues associated with increased industrialization of livestock farming.

In summary the literature suggests that farmers have concerns over the practicality and usefulness of PLF, whereas there is no information on the attitudes of other stakeholders toward these technologies. Questions of societal acceptability of PLF and chances to overcome these are only identified and discussed within conceptual approaches.

## Discussion

We aimed to investigate whether the literature allows for an answer to the question to which extent PLF has the potential to mitigate or reinforce societal concerns over current pig production. The papers reviewed present three main challenges regarding the societal acceptance of pig production: citizens' attitudes are rather negative, focus, and level of concerns are diverse, and there is a discordance between citizens' and farmers' evaluation of pig husbandry. However, in most studies participants evaluate predetermined items, which might bear the risk of adopting social conform stances and using existing frames and stereotypes (2). In addition, most surveys focus on concerns rather than on appreciation, which becomes evident from the often negative wording of the items, e.g., “The pigs feel comfortable in modern stables because they have no other experience” (25), instead of for instance “… because they are well cared for.” When phrasing their opinion freely, people raise similar aspects of concern but they also articulate which features of pig husbandry they appreciate, including automation (27). This limited evidence suggests that conventional pig production is not rejected *per se*, which may offer room for improvements within this system. It would be valuable to gain such qualitative insights into the attitudes of further stakeholders, for instance veterinarians. First results indicate that they share perspectives of both citizens and pig farmers (21), and might therefore take on a bridging role in the public debate.

If PLF is considered as means to tackle the challenges identified above, the following questions will arise: can PLF provide an answer to societal criticism; can it address the diversity of concerns, and can it bring citizens and farmers and their visions of pig husbandry closer together? These questions are difficult to answer. Except for one reference, PLF is no subject within the above-mentioned surveys on pig husbandry. One might argue that PLF is a relatively new technology and widely unknown outside agricultural and engineering communities. However, extensive research on PLF has been carried out in Europe since the past 20 years (37). At least less complex systems, such as automatic recognition and individual feeding of gestating sows are similarly common in practice as slatted flooring.

Regarding the literature on attitudes toward PLF in a broader context, there are two studies addressing farmers, i.e., the end-users of PLF, whereas there is a lack of information on the attitudes of other stakeholder groups, including citizens. Conceptual analyses of PLF by adopting a care ethics perspective, a parenting analogy or a biomimetic approach (33–35) may be difficult to translate to a wider public.

In this paper we show that it is not possible by means of the current body of literature to analyze whether PLF technologies can serve as an answer to public concerns related to pig production. First, there are no studies examining the attitudes of stakeholders toward PLF, except for farmers. This information is necessary to analyze whether PLF can play a role in addressing societal concerns. For the process of innovation this is crucial, as dealing with society implies more than providing information or knowledge, because that is insufficient to change (pre-)existing attitudes toward certain agricultural practices (14, 25). Second, we identified a tension between the evaluation of the problems to be addressed in pig production between citizen and farmers. Therefore, it is an omission that we lack sufficient information on the attitudes toward pig husbandry of those actors who might be able to mediate between different groups of society, such as veterinarians. Empirical data on public attitudes toward PLF and stakeholder views on pig production are essential elements for a comprised socio-ethical analysis. This combination of empirical data and normative analysis is a form of technology assessment that has already been applied in other contexts, such as in farming and food biotechnology (5). This approach enables on the one hand, to indicate and analyze potential objections against PLF that need further discussion and may result in further innovation. On the other hand, it helps to match appreciated aspects of PLF with current pig husbandry to address and deal with societal concerns.

## Author Contributions

MG and FM developed the concept and prepared the manuscript. All authors read, edited, and approved the final manuscript.

## Conflict of Interest

The authors declare that the research was conducted in the absence of any commercial or financial relationships that could be construed as a potential conflict of interest.
